# *In situ-*generated metal oxide catalyst during CO oxidation reaction transformed from redox-active metal-organic framework-supported palladium nanoparticles

**DOI:** 10.1186/1556-276X-7-461

**Published:** 2012-08-17

**Authors:** Jin Yeong Kim, Mingshi Jin, Kyung Joo Lee, Jae Yeong Cheon, Sang Hoon Joo, Ji Man Kim, Hoi Ri Moon

**Affiliations:** 1 Interdisciplinary School of Green Energy and KIER-UNIST Advanced Center for Energy, Ulsan National Institute of Science and Technology (UNIST), UNIST-gil 50, Ulsan, 689-798, Republic of Korea; 2 Department of Chemistry, BK21 School of Chemical Materials Science and Department of Energy Science, Sungkyunkwan University, Suwon, 440-746, Republic of Korea; 3 School of Nano-Bioscience and Chemical Engineering, KIER-UNIST Advanced Center for Energy and Low Dimensional Carbon Materials Center, Ulsan National Institute of Science and Technology (UNIST), Ulsan, 689-798, Republic of Korea

**Keywords:** redox reaction, metal-organic framework, CO oxidation, metal oxide, nanoparticle, palladium

## Abstract

The preparation of redox-active metal-organic framework (*ra*-MOF)-supported Pd nanoparticles (NPs) via the redox couple-driven method is reported, which can yield unprotected metallic NPs at room temperature within 10 min without the use of reducing agents. The Pd@*ra*-MOF has been exploited as a precursor of an active catalyst for CO oxidation. Under the CO oxidation reaction condition, Pd@*ra*-MOF is transformed into a PdO_*x*_-NiO_*y*_/C nanocomposite to generate catalytically active species *in situ*, and the resultant nanocatalyst shows sustainable activity through synergistic stabilization.

## Background

Metal-organic frameworks (MOFs) constitute an important class of porous crystalline materials. Modular synthetic routes enable rational design of MOFs with precise control over pore size, connectivity, and functional groups [1]. MOFs have widespread applications, including adsorption and separation [2-4], catalysis [5-8], and sensing [9,10]. In particular, MOFs are highly preferred for catalytic applications because of their large surface area and pore volume, tunable pore size and shape, and flexibility for diverse functionalization. As such, MOF-based catalysis has recently emerged as a burgeoning subfield in heterogeneous catalysis [2-4]. The active metal sites and/or reactive organic groups that constitute the frameworks of MOFs endow the catalytic functions of the MOFs. In addition, catalytic metal nanoparticles (NPs) incorporated into the cavities of MOFs can also provide catalytic function. The incorporation of metal NPs into porous supports, such as zeolites, mesoporous materials, and MOF, can be achieved by various methods, including solution impregnation [11-13], chemical vapor deposition [14-16], and solid grinding [17]. Although these methods have long been useful for generating heterogeneous catalysts, the high-temperature heating steps involved in these methods inevitably yield a wide distribution of particle sizes [18,19]. Another prominent route to the supported catalysts is by a colloidal deposition method, where pre-synthesized, highly monodisperse colloidal NPs are deposited onto the supports [20,21]. However, for full catalytic utilization of NP surfaces, this method requires judicious thermal or chemical treatments that can remove surface capping polymers or surfactants that stabilize colloidal NPs.

Recently, the conversion of the MOF into metal oxide nanoparticles has been used as a new strategy for preparing nanoscale functional entities. In this method, the secondary building units of MOF that are mostly composed of metal oxide clusters in angstrom scale were transformed into metal oxide nanomaterials by thermal treatments. For instance, Xu et al. reported the synthesis of Co_3_O_4_ nanoparticles converted from cobalt oxide subunits in a cobalt-based MOF, Co_3_(NDC)_3_(DMF)_4_ (NDC = 2,6-naphthalene-dicarboxylate; DMF = *N,N’*-dimethyl formamide) by pyrolysis in air [22]. The resulting cobalt oxide nanoparticles were utilized for an electrode material for lithium-ion batteries. More recently, MOF-5 was treated at high temperature (over 600°C) under various atmospheric conditions to produce ZnO nanoparticles and ZnO@C hybrid composites [23]. Thermal treatment of MOF-5 under nitrogen generated ZnO@C, whereas MOF-5 heated under air yielded a pure ZnO nanoparticle, indicating the combustion of organic ligands.

**Figure 1:**
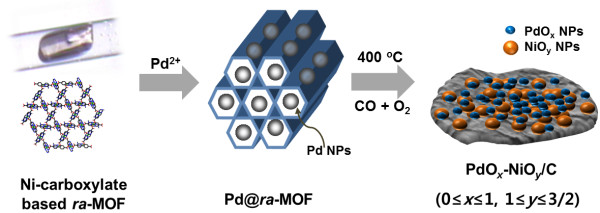
**Schematic representation for *in situ *formation of catalytically active species during CO oxidation reaction.** The reaction involves thermal transformation of crystalline MOF to nanoparticles in the solid state.

As an alternative scheme to the conversion of MOFs into new nanomaterials, one of the authors previously reported the redox couple-driven method where redox potential differences between foreign metal ions and redox-active MOFs (*ra*-MOFs) lead to the spontaneous formation of monodisperse metal NPs [24-26]. Significantly, the redox potential-driven method can readily yield highly monodisperse, surface-naked metal NPs at room temperature without the help of any reducing agent or surface capping molecules, which would be advantageous for catalytic applications. The application of this method for heterogeneous catalytic reactions, however, has not yet been exploited. In this study, we prepared Ni-based *ra*-MOF-supported Pd nanoparticles (Pd@*ra*-MOF) via the redox couple-driven method (Figure 1). We found that the Pd@*ra*-MOF transformed into PdO_*x*_-NiO_*y*_/C nanocatalyst during gas phase CO oxidation reaction. The resulting metal oxide nanocomposite showed high and sustainable catalytic activity toward CO oxidation.

## Methods

### Materials and characterization of samples

All chemicals and solvents used in the syntheses were of reagent grade and they were used without further purification. Infrared spectra were recorded with a Thermo Fisher Scientific Nicolet 6700 FT-IR spectrophotometer (Thermo Fisher Scientific, Waltham, MA, USA). Elemental analyses were performed at the UNIST Central Research Facilities Center in Ulsan National Institute of Science and Technology. Palladium content on *ra*-MOF was analyzed with an inductively coupled plasma optical emission spectrometer (Varian 720-ES, Varian Inc., Palo Alto, CA, USA). X-ray photoelectron spectroscopy was performed using a Thermo Scientific K-Alpha XPS spectrometer. X-ray diffraction (XRD) patterns were recorded with a Rigaku D/MAZX 2500 V PC diffractometer (Rigaku Corporation, Tokyo, Japan) at 40 kV and 100 mA with Cu-Kα radiations (1.54059 Å) with a scan speed of 2°/min and a step size of 0.02° in 2*θ* at room temperature. JEOL JEM-2100 F transmission electron microscope (JEOL Ltd., Akishima, Tokyo, Japan) and an Oxford INCA EDS unit (Oxford Instruments, Abingdon, Oxfordshire, UK) were used to examine the morphology of nanostructured catalysts before and after catalytic reactions. Thermogravimetric analysis (TGA) was performed under N_2_(g) atmosphere at a scan rate of 5°C/min using Q50 from TA instruments (New Castle, DE, USA). N_2_ sorption isotherms of *ra*-MOF and Pd@*ra*-MOF were obtained by BELSORP-max (BEL Japan Inc., Osaka, Japan) at 77 K to estimate the specific Brunauer-Emmett-Teller (BET) surface areas.

### Preparation of *ra*-MOF

[Ni(C_10_H_26_N_6_)](ClO_4_)_2_ (C_10_H_26_N_6_ = L_CH3_) was prepared according to the preparation conditions in a previous report [27]. [NiL_CH3_(bpdc) (*ra*-MOF; bpdc^2−^ = 4,4′-biphenyldicarboxylate) was synthesized by the modified method from the previous reports [24]. Synthetic detail is as follows: (NiL_CH3_)(ClO_4_)_2_ (0.80 g, 1.64 mmol) was dissolved in water/pyridine (48 mL, 2:1 *v*/*v*), and an aqueous solution (16 mL) of Na_2_bpdc (0.56 g, 2.10 mmol) was added. The solution was stirred over 20 min at room temperature, and 150 mL of methanol was added to it. The mixture was stirred for 6 h, forming pale purple microcrystalline precipitates which were isolated by filtration, washed with methanol, and dried in air. The as-prepared metal-organic framework was desolvated at 120°C under vacuum for 3 h resulting in a purple color. The yield is 57%.

### Preparation of the Pd@*ra*-MOF

The desolvated solid (0.43 g, 0.81 mmol) was immersed in 3.12 × 10^−2^ M acetonitrile solution (82 mL) of Pd(NO_3_)_2_· × H_2_O at room temperature and hand-shaken for 10 min, which resulted in a light brown solid. The resulting light brown powder was isolated by filtration, washed with acetonitrile, and dried in air.

### Catalytic activity test

The catalytic tests for CO oxidation were performed in a fixed bed reactor at atmospheric pressure, containing 0.06 g of catalyst samples. A feed mixture, prepared using mass flow controllers (MKS Instruments, Inc., Wilmington, MA, USA), contained 3.0% CO and 8.5% O_2_ and was balanced with He. The total flow rate of the feed mixture was 52 mL min^−1^, and the gas hourly space velocity was 1,7316 h^−1^. The effluent gas stream from the reactor was analyzed online by the thermal conductivity detector of parallel gas chromatography (Younglin Instrument Co., Ltd, Anyang, Korea) with a Carboxen 1000 column. In order to determine the conversion, the products were collected during 40 min of steady-state operation at each temperature. The empty reactor (without catalyst) showed no activity under identical conditions.

## Results and discussion

[NiL_CH3_(bpdc), which is composed of Ni(II) hexaaza macrocycle and carboxylate and has one-dimensional channels with a 7.3-Å pore opening (Additional file 1: Figure S1), was selected as the *ra*-MOF [24]. It has previously been shown that the MOF constructed by Ni(II) macrocyclic complexes and multidentate carboxylate ligands exhibits redox-active properties that originate from the six-coordinated Ni(II) sites [24-26]. The *ra*-MOF, [NiL_CH3_(bpdc) (bpdc = 4,4′-biphenyldicarboxylate), was prepared by the self-assembly of [NiL_CH3_(ClO_4_)_2_ and Na_2_bpdc in a H_2_O/pyridine mixture, yielding [(NiL_CH3_)_3_(bpdc)_3_·2pyridine·6H_2_O, and it was subsequently activated at 120°C under vacuum for 3 h. Pd NPs were spontaneously formed in the *ra*-MOF solid - without the use of any reducing agent - by soaking the *ra*-MOF in an acetonitrile solution of Pd(NO_3_)_2_ at room temperature for 10 min. Because the oxidation potential of Ni(II) to Ni(III) in the monomacrocyclic complexes ranges from 0.90 to 0.93 V [28], the redox reaction of a *ra*-MOF possessing Ni(II) macrocyclic complexes with Pd ions leads to the simultaneous oxidation of Ni(II) to Ni(III) and the reduction of Pd(II) ions to the metallic Pd NPs.

**Figure 2:**
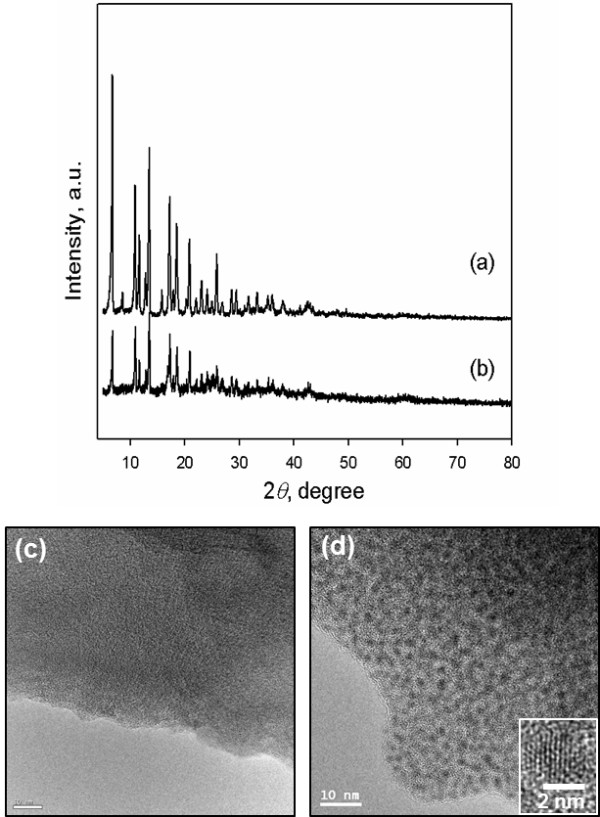
**XRD patterns and TEM images of (a, c) *ra*-MOF, and (b, d) Pd@*ra*-MOF, respectively.**

Figure 2 (a) and (b) display the XRD patterns of the as-synthesized *ra*-MOF and Pd@*ra*-MOF. The XRD peak intensities of Pd@*ra*-MOF decreased compared to those of the as-synthesized *ra*-MOF, which are presumably due to the partial occupation of Pd NPs in the pores of the *ra*-MOF as well as the partial destruction of the crystalline framework of the *ra*-MOF. This change was also observed with N_2_ adsorption measurements. The specific BET surface areas calculated from N_2_ adsorption decreased from 462 to 14 m^2^g^−1^ upon the formation of Pd NPs in the *ra*-MOF (Additional file 1: Figure S2). No characteristic peaks of Pd metal appeared in the XRD pattern of Pd@*ra*-MOF, which indicated that small-sized Pd NPs were well dispersed throughout the *ra-*MOF support. The *ra*-MOF and Pd@*ra*-MOF were further characterized by transmission electron microscopy (TEM) (Figure 2 (c) and (d)) and energy-dispersive X-ray spectroscopy (EDS) (Additional file 1: Figure S3). The TEM image clearly showed that uniform small Pd NPs that were 1.8 ± 0.3 nm in diameter were well dispersed on the *ra*-MOF. A high-resolution TEM image (Figure 2 (d), inset) showed the lattice fringes of Pd, indicating the single crystalline nature of Pd NPs. As determined by inductively coupled plasma analysis, the loading of Pd metal in the *ra*-MOF was calculated to be 3.6 wt.%.

**Figure 3:**
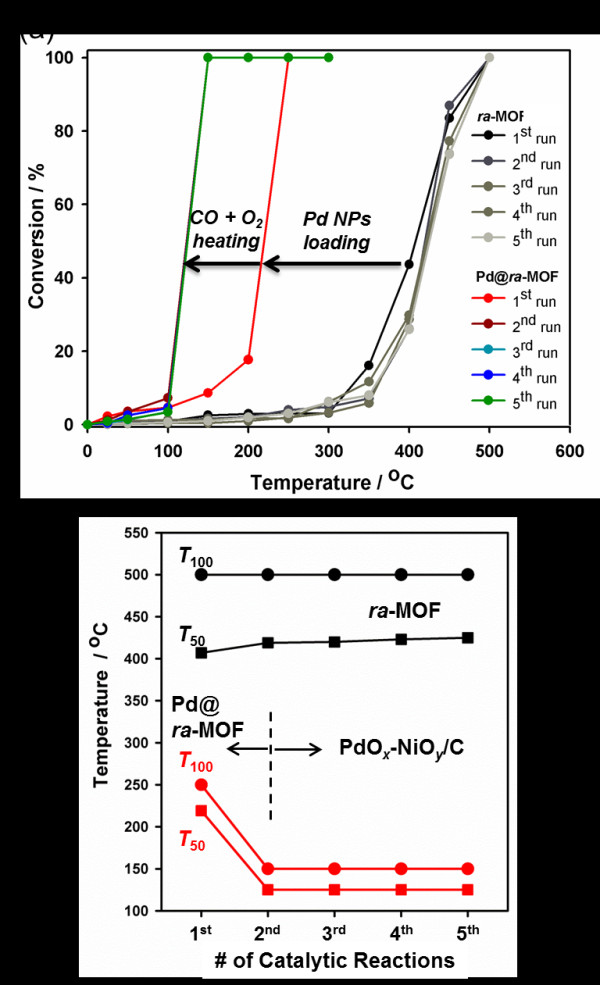
**Catalytic activities and conversion temperatures.** (**a**) Catalytic performance for CO oxidation of the as-prepared *ra*-MOF and Pd@*ra*-MOF. (**b**) Comparison of *T*_100_ and *T*_50_ for *ra*-MOF and Pd@*ra*-MOF according to the number of cycles.

The catalytic properties of Pd@*ra*-MOF were explored using CO oxidation as a probe reaction, and the as-prepared *ra*-MOF without Pd NPs was also tested for comparison. The catalytic oxidation of CO to CO_2_ has long been a benchmark reaction in heterogeneous catalysis; it is a continuous subject of fundamental mechanistic studies as well as of practical importance in many industrial processes, including the reduction of CO in automobile exhaust gases and the selective oxidation of fuel streams for polymer electrolyte fuel cells [29-31]. CO oxidation was performed in a fixed bed flow reactor at elevated temperatures under atmospheric pressure in a mixed gas composed of 3.0% CO, 8.5%O_2_, and 88.5% He. As shown in Figure 3, the as-synthesized *ra*-MOF exhibited poor catalytic activity, which is evidenced by a very high CO conversion temperature, with *T*_50_ and *T*_100_ (temperatures for 50% and 100% conversion, respectively) being 364°C and 500°C, respectively. Successive four runs showed similar catalytic behaviors. In contrast, the loading of Pd NPs into *ra*-MOF resulted in markedly lower conversion temperatures of *T*_50_ and *T*_100_ at 222°C and 250°C, respectively, indicating the Pd@*ra-*MOF had enhanced catalytic activity over the *ra*-MOF for CO oxidation. The catalytic abilities of Pd@*ra*-MOF in the first run, however, are not superior to other Pd catalysts, and this probably comes from the sharp decrease of surface area after formation of larger-sized Pd NPs than the channel size of *ra*-MOF, which lessens the accessibility of CO and O_2_ to Pd NPs embedded in *ra*-MOF. The CO oxidation activities over Pd@*ra*-MOF were repeatedly tested, and the Pd@*ra*-MOF exhibited further lowered conversion temperatures in the second run, with *T*_50_ and *T*_100_ dropping down to 125°C and 150°C, respectively, thus revealing significantly enhanced CO oxidation activity as compared to the first run (Figure 3). This implied the formation of new species from the *ra*-MOF and Pd NPs during the first catalytic reaction. As shown in Figure 3, three more reaction runs over the Pd@*ra*-MOF produced the same results as the second run, demonstrating its long-term stability.

**Figure 4:**
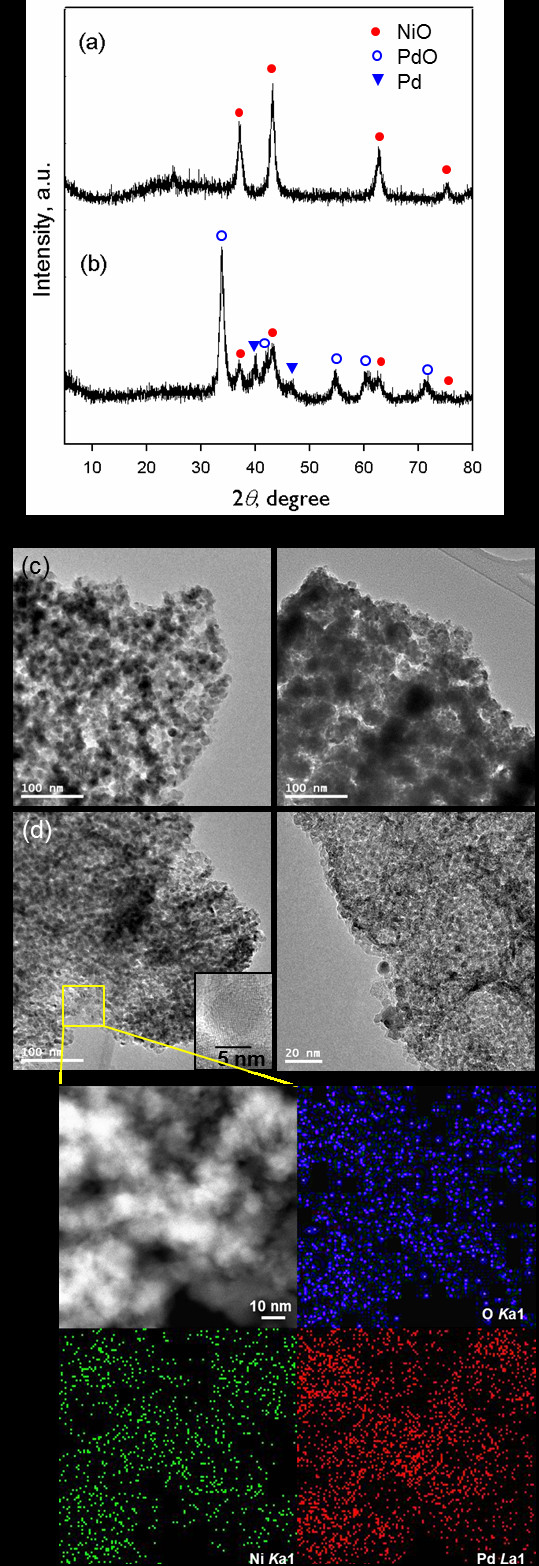
**XRD patterns, TEM images, and EDS mapping.** XRD patterns and TEM images of (a, c) *ra*-MOF and (b, d) Pd@*ra*-MOF, respectively, after CO oxidation reactions. (e) EDS mapping of Pd@*ra*-MOF after CO oxidation reaction.

**Figure 5:**
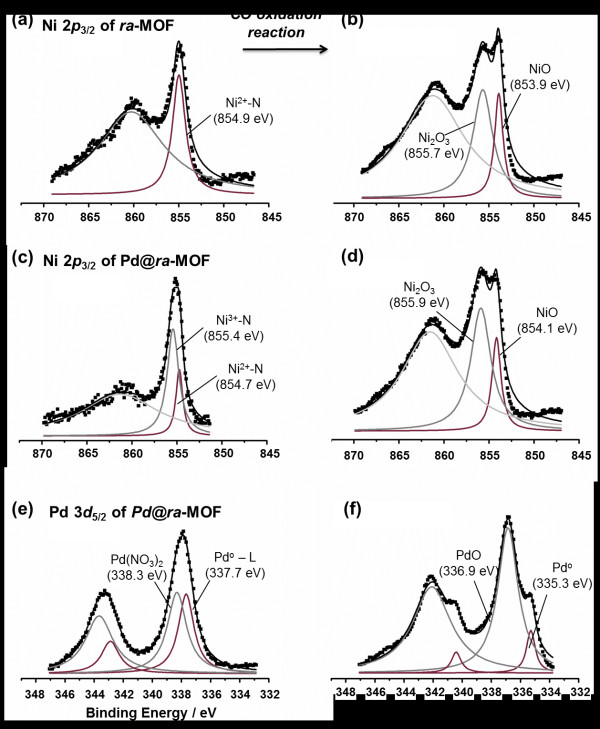
**XPS spectra.** (**a**) Ni 2*p*_3/2_ of *ra*-MOF, (**c**) Ni 2*p*_3/2_ of Pd@*ra*-MOF, and (**e**) Pd 3*d*_5/2_ of Pd@*ra-*MOF before and (**b**, **d**, **f**) after CO oxidation reaction (fifth run).

To examine the transformation of the Pd@*ra*-MOF during a CO oxidation reaction, the XRD patterns and TEM images of the as-synthesized *ra*-MOF and Pd@*ra*-MOF after catalytic reactions were obtained and compared (Figure 4). The XRD pattern of the *ra*-MOF after the fifth run of the catalytic reactions (Figure 4 (a)) was completely different from that of the as-prepared *ra*-MOF (Figure 2 (a)). The diffraction peaks were assigned as cubic NiO of the Fm3^-m space group (JCPDS 47–1049). It appears that during CO oxidation, building blocks of *ra*-MOF (hexaaza macrocyclic ligands, Ni ions, and bpdc^2−^ ligands) were transformed into different species under oxidative reaction conditions. Thermally unstable hexaaza macrocyclic ligands, L_CH3_, coordinating Ni ions in the *ra*-MOF could be easily decomposed and removed during a high-temperature catalytic reaction. This was substantiated by the X-ray photoelectron spectroscopy (XPS) of the *ra*-MOF, whose nitrogen content approached to nearly zero after the catalytic reaction (Additional file 1: Figure S4). Ni ions were oxidized to form NiO and Ni_2_O_3_ species under an oxidative reaction environment, which was proven by Ni 2*p*_3/2_ XPS spectra that indicated the formation of Ni_2_O_3_ (855.7 eV) as well as NiO (853.9 eV) (Figure 5 (b)) from N-coordinating Ni(II) (854.9 eV) (Figure 5 (a)). Lastly, the organic ligand bpdc^2−^ was decomposed and converted to a carbogenic support by heating up to 500°C, as evidenced by a TGA trace (Additional file 1: Figure S5) and infrared (IR) spectra before and after catalytic reactions (Additional file 1: Figure S6). Overall, the resultant material after the catalytic reaction was a NiO_*x*_/C nanocomposite. The TEM image also revealed the formation of spherical crystalline NiO_*x*_ NPs on a carbogenic support (Figure 4 (c)). The size of the NiO NPs was estimated by applying the Debye-Scherrer equation to the (200) reflection at 2*θ* = 43.2° of the XRD pattern (Figure 4 (a)), and the derived diameter of crystalline NiO NPs was 9.8 nm, which was consistent with the size estimated by TEM.

A similar phenomenon occurred in the Pd@*ra*-MOF catalyst during CO oxidation. The XRD pattern of the catalyst after five consecutive CO oxidation reactions up to 300°C (Figure 4 (b)) indicated the formation of NiO and PdO as well as the presence of pre-existing Pd(0) species. The NiO species were formed by the thermal transformation of *ra*-MOF (as described above), and the PdO NPs were generated by the oxidation of metallic palladium during the reaction. The XRD peaks for NiO and PdO species were broad, and the crystalline sizes determined by the Scherrer equation were 8.9, 9.9, and 10.3 nm for NiO, PdO, and Pd, respectively. The TEM observation of Pd@*ra*-MOF before and after the CO oxidation (Figure 4 (d)) also confirmed the formation of spherical NiO and PdO nanoparticles. Contrary to the complete decomposition of *ra*-MOF at 500°C, the organic ligands, bpdc^2−^ in Pd@*ra*-MOF, were partially decomposed to carbogenic supports and partially intact at a relatively low temperature, 300°C as an IR spectrum showed peaks for *ν*_O-C=O_ at 1,593(s) cm^−1^ and *ν*_C=C(aromatic)_ at 1,528(s) cm^−1^, respectively (Additional file 1: Figure S7). As shown in Figure 4 (e), EDS mapping of the PdO_*x*_-NiO_*y*_/C catalyst after CO oxidation indicated that the PdO_*x*_ and NiO_*y*_ NPs were well dispersed on the carbogenic support. The morphology of the PdO_*x*_ NPs was maintained after repeated CO oxidation runs, as evidenced by TEM images (Figure 4 (d), right).

This might be attributed to the immobilization of the PdO_*x*_ and NiO_*y*_ NPs by carboxylate ligands coexisted on the carbogenic support and the consequent suppression of migration and aggregation of NPs, even under the harsh CO oxidation reaction condition.

Changes in the chemical states of Ni and Pd species in the Pd@*ra*-MOF before and after the catalytic reaction were monitored by XPS. Before the CO oxidation reaction, Pd@*ra*-MOF reasonably contained N-coordinating Ni(III) species (855.4 eV) as well as Ni(II) (854.7 eV), which were generated by the redox reaction with Pd(II) (Figure 5 (c)). However, after the CO oxidation reaction, Ni_2_O_3_ (855.9 eV) and NiO (854.1 eV) were formed by the thermal transformation of Ni(II/III) macrocyclic complexes, similar to the case of the as-prepared *ra*-MOF catalyst (Figure 5 (d)). Before the Pd@*ra*-MOF underwent a catalytic reaction, the small-sized Pd^0^ clusters (337.7 eV), which were strongly interacting with aromatic ligands of the *ra*-MOF [32], coexisted with unreduced Pd(NO_3_)_2_ species (338.3 eV) (Figure 5 (e)). After the reaction, most of the Pd species were oxidized to PdO (336.9 eV) under a highly oxidative reaction condition, while the rest remained in the reduced Pd^0^ state (335.3 eV). It is noteworthy that the binding energy of the latter was shifted to a lower energy level compared to that of Pd@*ra*-MOF before the catalytic reaction (Figure 5 (f)). This shift was due to the enlargement of the Pd NPs from approximately 2 nm to approximately 10 nm, which was consistent with the XRD results (Figure 4 (b)). Based on the XPS, XRD, and TEM results, the Pd@*ra*-MOF was transformed into the PdO_*x*_-NiO_*y*_/C nanocomposite (0 ≤ *x* ≤ 1, 1 ≤ *y* ≤ 3/2) catalyst during the catalytic reaction, where each species showed the synergetic catalytic effect to convert CO to CO_2_ at a very low temperature. In addition, the PdO_*x*_-NiO_*y*_/C catalyst after the fifth run showed a higher BET surface area (35 m^2^/g) than that of Pd@*ra*-MOF, which can result in improved catalytic performance by providing active sites for catalyzing surface reaction (Additional file 1: Figure S2). However, the surface area is still very low, which implies that the significant portion of active sites of PdO and NiO NPs can be buried and covered by carbon support. This might result in the loss of catalytic activity from the potentially expected.

The various metal oxides supporting metal NP catalysts have been investigated as CO oxidation reaction catalysts. Machida et al*.* reported the CO oxidation activity of metallic Pd NPs supported on CeO_2_, which was significantly enhanced by thermal aging of the catalyst [33]. It was revealed that the thermal treatment caused the strong metal-support interaction via Pd-O-Ce bonding, which prevented the sintering of Pd oxide species at high temperature and promoted CO adsorption to react with oxygen. Haruta and other groups studied on supported gold catalysts on metal oxides such as TiO_2_, Fe_2_O_3_, Al_2_O_3_, CuO, La_2_O_3_ NiO, and Y_2_O_3_[34-36]. The activation of O_2_ molecules has been shown to occur at the perimeter between Au NPs and the metal oxide support, highlighting the importance of metal-metal oxide interface in promoting CO oxidation [37]. In this study, the catalytically active species for the CO oxidation reaction, PdO_*x*_-NiO_*y*_/C nanocomposite, was generated *in situ* in the reaction environment, and its stable, sustainable activity under cycled conditions could be ascribed to the synergistic interaction among the PdO, NiO NPs, and carbogenic supports. In addition, compared to the conventional methods, our *in-situ* generation method of MO_*x*_@C catalysts has an advantage due to the simple preparation procedure which can provide a strong metal-metal oxide interaction as well as carbon-metal oxide interaction during the reaction. In general, MO_*x*_@C catalysts are synthesized by loading metal precursors on carbon supports and successive pyrolysis, which is mostly conducted at high temperature for a long time [38]. In the present work, the spontaneously formed strong interactions not only provide the enhancement of catalytic properties, but also reduce mobility of metal precursors on the carbon and metal surfaces, which results in small and monodispersed metal oxide nanocrystals.

## Conclusions

In conclusion, we reported the preparation of *ra*-MOF-supported Pd NPs, Pd@*ra*-MOF, via the redox couple-driven method, which yields unprotected metallic NPs at room temperature within a few minutes without the use of reducing agents. We found that during the CO oxidation reaction, the Pd@*ra*-MOF was transformed into a PdO_*x*_-NiO_*y*_/C nanocomposite that showed sustainable and enhanced catalytic activity through the synergistic stabilization of catalytically active PdO species. This study creates new opportunities for taking advantage of the MOF’s vulnerable point to develop a novel class of heterogeneous catalysts by utilization of MOFs or metal NPs@MOF as precursors.

## Competing interests

The authors declare that they have no competing interests.

## Authors’ contributions

JYK, MJ, KJL, JYC carried out the synthetic experiments and characterizations. SHJ, JMK, HRM conceived the study, participated in its design and coordination, and drafted the manuscript. All authors read and approved the final manuscript.

## Supplementary Material

Additional file 1 **Supplementary information.** A file showing seven supplementary figures for the *in situ*-generated metal oxide catalyst during CO oxidation reaction. Click here for file
