# Supplementary material for: In situ-generated metal oxide catalyst during CO oxidation reaction transformed from redox-active metal-organic framework-supported palladium nanoparticles
Source: Nanoscale Res Lett. 2012 Aug 17;7(1):461. doi: 10.1186/1556-276X-7-461 (PMC3492126; doi:10.1186/1556-276X-7-461)
Supplement: Additional file 1 — Supplementary information. A file showing seven supplementary figures for the in situ-generated metal oxide catalyst during CO oxidation reaction. [file 1556-276X-7-461-S1.pdf]

Supporting Information

In situ-generated metal oxide catalyst during CO oxidation reaction transformed from redox-active metal-organic framework supported palladium nanoparticles

Jin Yeong Kim,[a] Mingshi Jin,[b] Kyung Joo Lee,[a] Jae Yeong Cheon,[c] Sang Hoon Joo,[c] Ji Man Kim,[b]* and Hoi Ri Moon[a]**

[a] *Interdisciplinary School of Green Energy, Ulsan National Institute of Science and Technology (UNIST), Ulsan 689-798, Republic of Korea. E-mail: hoirimoon@unist.ac.kr*
Fax: +82-52-217-2019; Tel: +82-52-217-2928

[b] *Department of Chemistry, BK21 School of Chemical Materials Science and Department of Energy Science, Sungkyunkwan University, Suwon 440-746, Republic of Korea.E-mail:jimankim@skku.edu*
Fax: +82-31-290-7075; Tel: +82-31-290-7074

[c] *School of Nano-Bioscience and Chemical Engineering, Ulsan National Institute of Science and Technology (UNIST), Ulsan 689-798, Republic of Korea. E-mail: shjoo@unist.ac.kr*
Fax: +82-52-217-2019; Tel: +82-52-217-2522

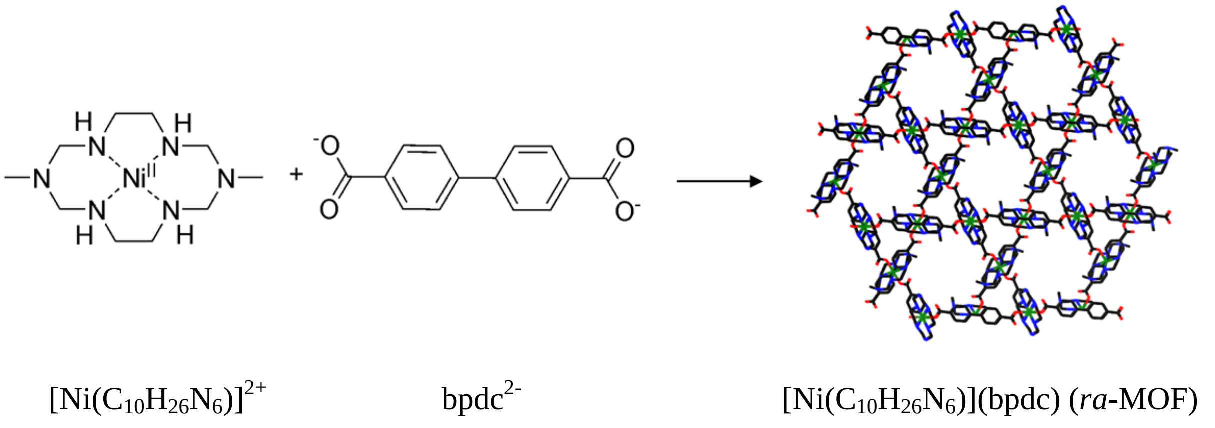

Figure S1. Synthetic scheme of *ra*-MOF by self-assembly of $[Ni(C_{10}H_{26}N_6)]^{2+}$ and $bpdc^{2-}$

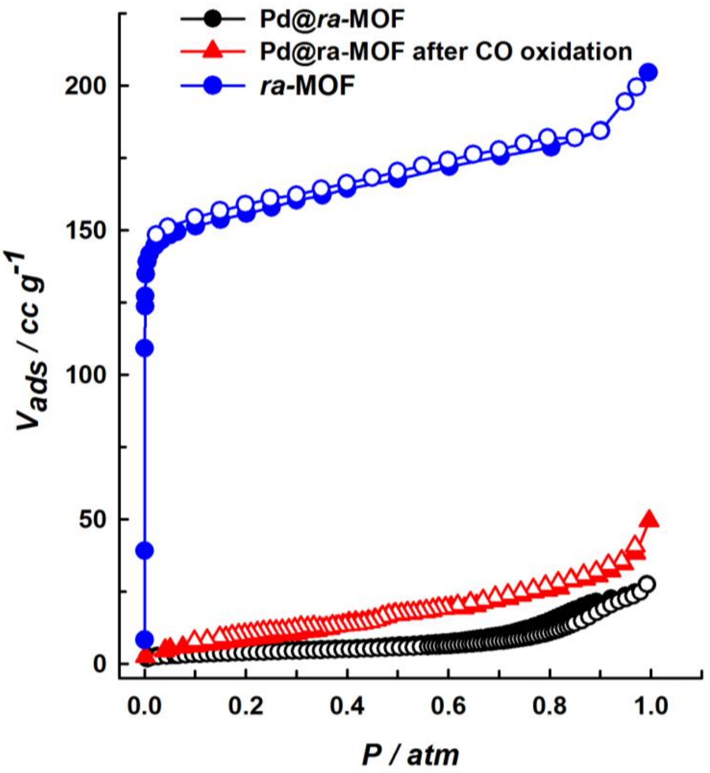

Figure S2. Comparison of N_2 sorption data for *ra*-MOF and Pd@*ra*-MOF before and after CO oxdiation reaction

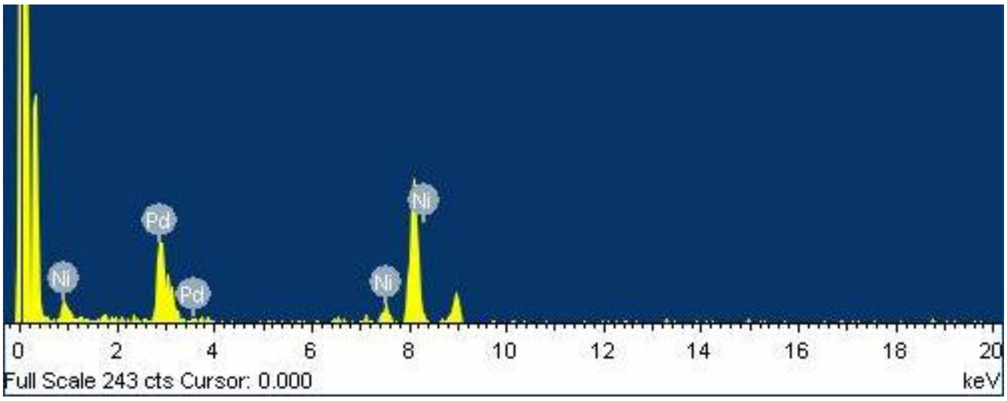

Figure S3. EDS result of Pd@*ra*-MOF

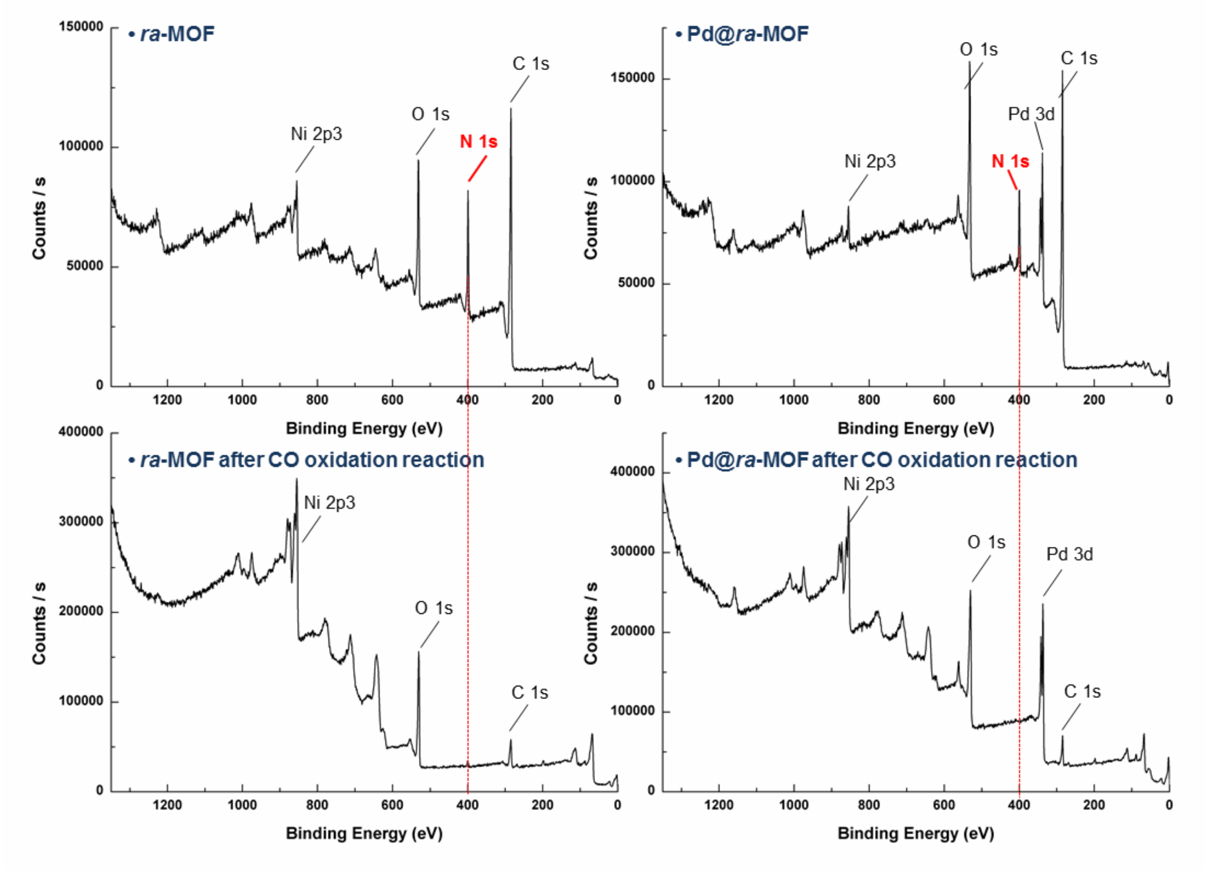

Figure S4. XPS wide scan spectra for *ra*-MOF and Pd@*ra*-MOF before and after CO oxidation reaction, respectively.

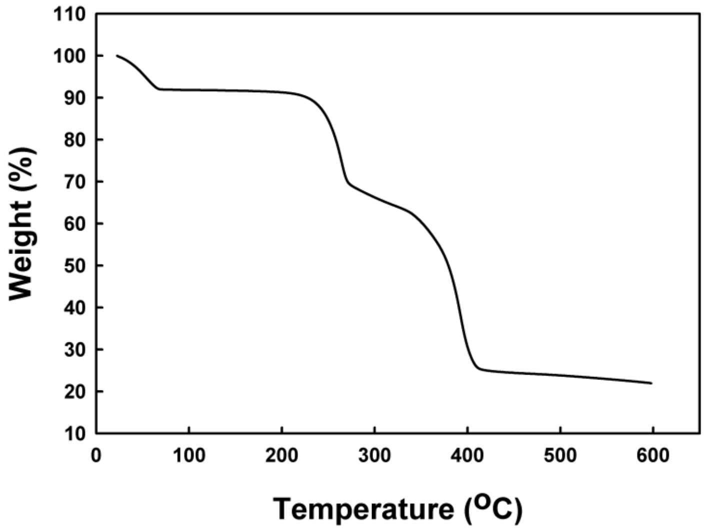

Figure S5. TGA trace of *ra*-MOF

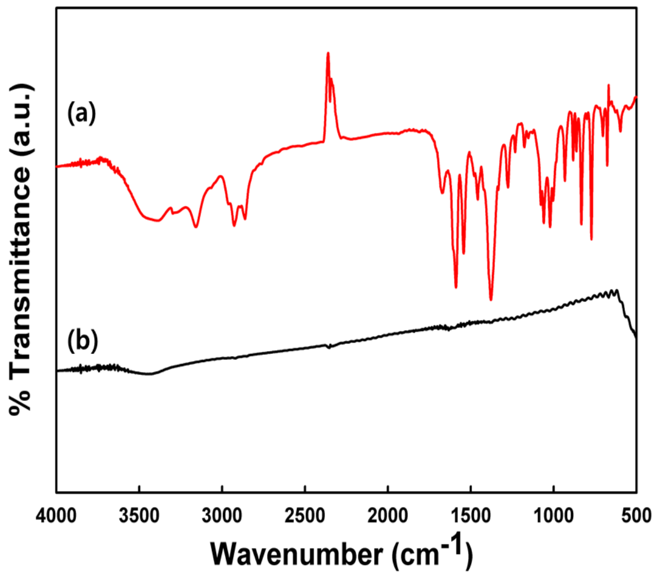

Figure S6. Comparison of IR spectra for *ra*-MOF (a) before and (b) after CO oxdiation reaction upto 500 ^{o}C.

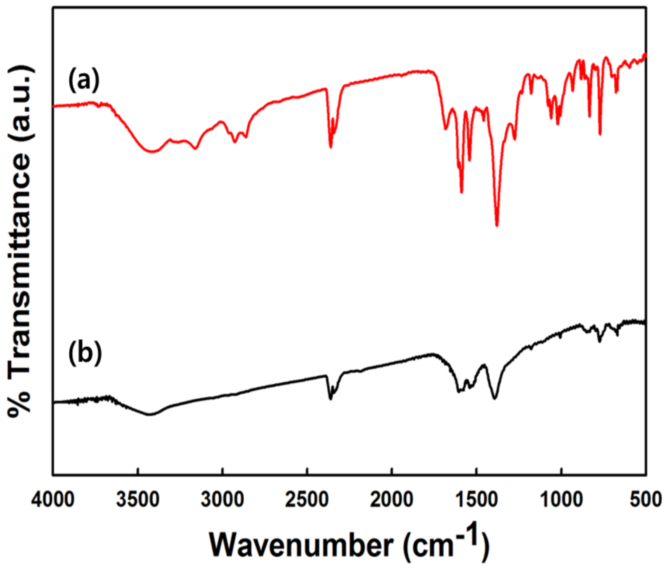

Figure S7. Comparison of IR spectra for Pd@*ra*-MOF (a) before and (b) after CO oxdiation reaction upto 300 ^{o}C.
